# Effects of Pig Dietary n-6/n-3 Polyunsaturated Fatty Acids Ratio and Gender on Carcass Traits, Fatty Acid Profiles, Nutritional Indices of Lipid Depots and Oxidative Stability of Meat in Medium–Heavy Pigs

**DOI:** 10.3390/foods12224106

**Published:** 2023-11-12

**Authors:** Giovanna Minelli, Katia D’Ambra, Paolo Macchioni, Domenico Pietro Lo Fiego

**Affiliations:** 1 Department of Life Sciences (DSV), University of Modena and Reggio Emilia, Via G. Amendola 2, 42122 Reggio Emilia, Italy; domenicopietro.lofiego@unimore.it; 2 Interdepartmental Research Centre for Agri-Food Biological Resources Improvement and Valorisation (BIOGEST-SITEIA), University of Modena and Reggio Emilia, P. le Europa, 1, 42124 Reggio Emilia, Italy; 3 Department of Agricultural and Food Sciences (DISTAL), University of Bologna, Viale G. Fanin 46, 40127 Bologna, Italy; paolo.macchioni@unibo.it

**Keywords:** pig lipids, dietary n-6/n-3 PUFA ratio, gender, carcass traits, fatty acid profile, oxidative stability, lipid nutritional indices

## Abstract

The effects of different dietary n-6/n-3 polyunsaturated fatty acids (PUFA) ratios and gender on key carcass traits, as well as the nutritional and technological quality of lipids in medium–heavy pig tissues have been poorly studied. To investigate the subject, 24 Large White, barrows and gilts, evenly divided into two groups of 12, were fed from 80 kg of live-weight (LW) until slaughter at 150 kg LW, either a high (9.7:1) (HPR) or low (1.4:1) (LPR) dietary n-6/n-3 PUFA ratio. On individual samples of longissimus thoracis muscle (LTM), subcutaneous (SF) and perirenal (PF) adipose tissues (ATs), the fatty acid (FA) composition was determined by gas chromatography, and lipid nutritional indices (LNIs) were calculated. The oxidative stability of meat was evaluated by determining the malondialdehyde content on raw and cooked (24 h postmortem) and refrigerated (8 days postmortem) LTM samples. The carcass traits did not vary between genders and diets. The LPR group showed a higher n-3 PUFA level and a lower n-6/n-3 PUFA ratio in all the tissues examined and better LNI, especially in the ATs. Diet did not affect the oxidative stability of meat. Gender did not influence the n-6/n-3 PUFA ratio, while barrows showed improvements in some LNI in ATs. Reducing the n-6/n-3 ratio in the diet of growing–finishing medium–heavy pigs improved the FA profile in all tissues and most LNI in ATs without impairing the oxidative stability of meat.

## 1. Introduction

Nowadays, consumers are paying increasing attention to the nutritional quality and health benefits of what they eat. In particular, the lipid amount and characteristics in foods go under unprecedented scrutiny. It is well known that some dietary saturated fatty acids (SFAs) increase total serum LDL-cholesterol concentrations [1,2,3], while polyunsaturated fatty acids (PUFAs) have a hypocholesterolemic effect [4]. Additionally, PUFAs, most notably those of the n-6 and n-3 series, exert several beneficial effects on human health and are essential for many physiological functions. In particular, n–6 PUFAs show an antiatherogenic action, and n–3 PUFAs are known for their antithrombogenic effect [5] and their correlation with a low incidence of cardiovascular diseases and atherosclerosis [6,7,8,9,10,11]. Intriguingly, the ratio between n-3 and n-6 PUFAs seems to play itself an important role, with several evidence indicating that diets with high n-3 PUFA content and low n-6/n-3 PUFA ratio are more beneficial to human health [12,13]. However, the typical Western diet presents a n-6/n-3 PUFA ratio that ranges from 15:1 to 20:1 [14,15], while the recommended ratio varies between 1:1 and 4:1 [16]. In the light of the above, the content of individual fatty acids (FAs) and the ratio between FA classes are used to calculate common lipid nutritional indices (LNIs), used to evaluate nutritional quality and healthiness of the lipid profile of food. Monitoring, understanding, and tuning the FA profile in meat and related products is thus key for quality control and impact on human health.

Meat and meat products are essential components of a balanced diet given their content of amino acids, vitamins, minerals, and FAs [17]. Nonetheless, they are seen as potentially unhealthy foods, mainly because of their high content of SFAs, cholesterol, and high n-6/n-3 PUFA ratio [18]. Therefore, much attention is paid to their FA profile, and there is a strong interest in modifying their FA composition toward a more favorable n-6/n-3 PUFA ratio. In pigs, as in other monogastric animals, the FA composition of intramuscular fat (IMF) and adipose tissues (ATs) depends on many factors such as genetics [19,20,21], gender, age, live weight (LW) at slaughter [22,23,24,25] and feeding practices [26,27,28]. For example, at the same LW, lipids in entire males are more unsaturated than in females, while castrated males, which yield more adipose carcasses, show a higher degree of saturated fatty acids [22,29]. As would be expected, total carcass fatness and SFA content increase with increasing LW [23,29]. In addition, pigs cannot convert dietary n-6 PUFAs into n-3 PUFAs due to the lack of the n-3 FA desaturase gene; the most common approach to improve n-6/n-3 PUFA ratio in pork meat is thus providing a high content of n-3 PUFAs in the diet of pigs.

The inclusion of flaxseed in pig diets has been successfully exploited to this end [30,31,32,33,34], and the results have shown that it is a suitable means to increase the n-3 PUFA content and to reduce the n-6/n-3 PUFA ratio in pork. However, most of the studies have involved lightweight pigs, and some parameters, such as gender and LNIs of Ats, are often overlooked. Very limited data are available on medium–heavy pigs [35]. This class of pigs, slaughtered at a LW of 130–150 kg, is gaining more and more importance in the Italian pig industry because of better feed conversion compared to heavier pigs and because they are not subject to the limitations imposed by the Protected Denomination of Origin (PDO) rules. For this reason, further research on the effect of lowering the dietary n-6/n-3 PUFA ratio in the feeding of medium–heavy pigs is needed. However, a further concern is that the higher degree of unsaturation in meat and meat products has been shown to reduce their shelf life, due to increased lipid oxidation [30,36,37].

This study aimed to investigate the effect of gender and n-6/n-3 PUFA ratio in the grower–finisher diet on key carcass traits, the oxidative stability of raw and cooked meat, the FA profile, and the lipid nutritional indices (LNIs) of intramuscular (IMF), subcutaneous (SF) and perirenal (PF) adipose tissues (ATs) in medium–heavy pigs.

## 2. Materials and Methods

### 2.1. Livestock and Diets

The study was performed on 24 Italian Large White pigs intended for Italian medium–heavy pig production. The subjects balanced for gender (12 barrows and 12 gilts) and live weight (average LW 79.9 ± 6.1 kg), were evenly housed in 8 concrete-floored pens of 9 m^2^ each, 3 animals per pen (4 replicates), and assigned to two dietary treatments differing for the n-6/n-3 PUFA ratio (PR). The composition of feeds is shown in Table 1. The high PR (HPR) barley/soybean diet had a PR value of 9.97:1 in the growing (80–113 kg LW) and 9.42:1 in the finishing (113–150 kg LW) periods, respectively, whereas in the low PR (LPR) diet, where 5% of extruded flaxseed was included, the PR ratio was equal to 1.47:1 and 1.24:1 in the same periods. The two diets were isoenergetic and isoproteic, and they had the same lysine/digestible energy ratio. Feed was administered at a rate of 7.5% of LW^0.75^ in the growing phase and 8.5% of LW^0.75^ in the finishing phase. Animals had ad libitum access to water. Pigs were slaughtered at an average LW of 149.9 ± 10.6 kg after 104 days of trial. All the experimental procedures performed in this study complied with the Italian legislation and did not require special animal care authorizations, according to the decision of the Animal Welfare Committee of Consiglio per la Ricerca in Agricoltura e l’Analisi dell’Economia Agraria (CREA; 14 September 2016), according to the Italian Legislative Decree 4 March 2014 n.26 art.2 point F.

### 2.2. Slaughtering and Sampling Procedures

After overnight fasting, the pigs were individually weighed, transported to a commercial abattoir according to the Council Regulation (EC) 1/2005 on the protection of animals during transport and related operations, and slaughtered by exsanguination after electrical stunning, in agreement with the Council Regulation (EC) No 1099/2009 on the protection of animals at the time of the killing. After slaughtering, each carcass was graded in agreement with EUROP grid carcass grading, using the Fat-o-Meat’er (Frontmatec, Kolding, Denmark) device [38]. Subsequently, each carcass was dissected into commercial cuts, and samples of longissimus thoracis muscle (LTM) and SF, both at the level of the last rib, and a sample of PF, were taken from each left half- carcass. All the samples were transported to the laboratory in a refrigerated box. Each sample of LTM was sliced into three subsamples: one for oxidative stability evaluation before and after the cooking procedure, a second subsample; the SF and PF samples were vacuum packed (Elegen, Reggio Emilia, Italy) and stored at −20 °C until the lipid extraction for FAs analyses. A third subsample from each LTM was packed in MAP (70% O_2_ and 30% CO_2_) using a high-barrier tray lidded with a PET/EVOH/PE film (AERPACK System, supplied by Coopbox Group, Italy), and stored in a refrigerator (4 ± 1 °C) for 8 days for oxidative stability evaluation after storage. The whole-package oxygen transmission rate (OTR) was <0.1 cm^3^ day^−1^ (air, 25 °C).

### 2.3. Oxidative Stability of Raw and Cooked Muscle

Oxidative stability was evaluated by the 2-thiobarbituric acid reactive substances (TBARS) measurements [27,39]. For the cooking procedure, slices of LTM of about 2.5 cm thickness and 100 g weight were individually vacuum packed in plastic bags and put in a water bath at a temperature of 80 °C, until the core temperature, controlled during cooking with a temperature probe, reached 70 °C. Then, slices were cooled under running water. Absorbance at 532 nm was measured against a blank sample on two replicates of each sample on a Jasco spectrophotometer (Model V550, UV/VIS, Tokyo, Japan) immediately after cooling. TBARS were expressed as mg of malondialdehyde (MDA) per kg of muscle using a tetraethoxypropane (TEP) (Sigma-Aldrich, Milan, Italy) as a standard.

### 2.4. Fatty Acid Profile

The total lipids of LTM, SF and PF were extracted according to the method of Folch, Lees, and Sloane Stanley [40]. In total, 50 mg of extract lipids were methylated before the gas-chromatographic analysis as detailed in previous papers [21,41]. The fatty acid methyl esters content (FAMEs) was expressed as g/100 g of total lipids.

### 2.5. Lipid Nutritional Indices (LNI) and Iodine Value (IV)

From the FA composition data, the total amounts of saturated FA (SFA), monounsaturated FA (MUFA), polyunsaturated FA (PUFA), unsaturated FA (UFA = MUFA + PUFA), n-6 PUFA, n-3 PUFA, n-3 long-chain PUFA (n-3 LCPUFA = eicosatrienoic (C20:3n-3)+ eicosapentaenoic (EPA, C20:5n-3)+ docosapentaenoic (DPA, C22:5n-3) + docosahexaenoic (DHA; C22:6n-3), essential FA (EFA= linoleic (LA) + αlinolenic (ALA)+γlinolenic (GLA) + arachidonic (C20:4n-6) and UFA/SFA, PUFA/SFA, and n-6/n-3 PUFA ratios were calculated. Moreover, some other nutritional indices as H/H [42], HPI [43], AI [44], TI [44], UI [45] and PI [46], were calculated according to the following equations:

Hypocholesterolemic/hypercholesterolemic ratio (H/H) = (C18:1n-9+ΣPUFA)/(C12:0 + C14:0 + C16:0) (1)

Health-promoting index (HPI) = ΣUFA/[C12:0 + (4 × C14:0) + C16:0] (2)

Atherogenic index (AI) = [C12:0 + (4 × C14:0) + C16:0]/ΣUFA (3)

Thrombogenic index (TI) = (C14:0 + C16:0 + C18:0)/(0.5 × ΣMUFA) + (0.5 × Σn-6 PUFA) + (3 × Σn-3 PUFA) + Σn-3 PUFA/Σn-6 PUFA) (4)

Unsaturation index (UI) = (% monoenoics × 1) + (% dienoics × 2) + (% trienoics × 3) + (% tetraenoics × 4) + (% pentaenoics × 5) + (% hexaenoics × 6) (5)

Peroxidizability index (PI) = (% monoenoics × 0.025) + (% dienoics × 1) + (% trienoics × 2) + (% tetraenoics × 4) + (% pentaenoics × 6) + (% hexaenoics × 8) (6)

On SF and PF fat samples, starting from the FA profile and according to the following formula [47], iodine value (IV) was calculated:

IV = (85.703 + [C14:0] × 2.740 − [C16:0] × 1.085 − [C18:0] × 0.710 + [C18:2n − 6] × 0.986) (7)

### 2.6. Statistical Analysis

The data were subjected to analysis of variance using the general linear model (GLM) procedure of SAS (SAS Institute Inc., Cary, NC, USA). The statistical model included, within each anatomical location (LTM, SF and PF), dietary treatment (HPR and LPR), gender (gilts and barrows) and their interactions as fixed effects. The interactions were not found to be statistically significant (*p* > 0.05) and therefore were not presented in the tables. A Principal Component Analysis (PCA) was performed to describe the relationship between the dietary treatments and the principal classes of fatty acids and nutritional indices of lipid depots. The PCA was performed using RStudio software, version 2023.03.0 + 386.

## 3. Results

### 3.1. Carcass Characteristics

Table 2 displays the effects of dietary treatment and gender on carcass traits, specifically comparing the hot carcass weight covariate for the slaughter live weight.

The gender and the different dietary n-6/n-3 PUFA ratio had no significant effect on any of the parameters measured after slaughter (*p* > 0.05).

### 3.2. Fatty Acid Profile, Lipid Nutritional Indices and Oxidative Stability of Longissimus Thoracis Muscle (LTM)

Table 3 displays the effects of dietary treatments and gender on the intramuscular fatty acid profile of the longissimus thoracis muscle.

The dietary treatment did not result in significant changes in the proportions of total SFA, MUFA or PUFA (*p* > 0.05).

In a more detailed analysis, pigs fed with a low dietary n-6/n-3 PUFA ratio of 1.4:1 exhibited decreased contents of heptadecenoic (C17:1) (*p* < 0.01), arachidonic (*p* < 0.05) and docosatetraenoic (C22:4n-6) (*p* < 0.01) FAs. Conversely, they displayed increased contents (*p* < 0.01) of ALA, eicosatrienoic, EPA and DPA fatty acids, resulting in higher (*p* < 0.01) proportions of total n-3 PUFA and n-3 LCPUFA, except for DHA (*p* > 0.05).

The contents of the remaining FAs in LTM were not significantly influenced by the dietary treatments (*p* > 0.05).

The gender did not substantially modify the FA composition of LTM except for lauric, palmitoleic (C16:1), vaccenic (C18:1n-7) and eicosenoic (C20:1) Fas, which were higher (*p* < 0.05) in barrows, while DHA was lower (*p* < 0.01) than in gilts.

Table 4 displays the effects of dietary treatment and gender on the lipid nutritional indices of intramuscular fat of LTM. The pigs that received the low n-6/n-3 PUFA dietary ratio (LPR group) showed a significantly (*p* < 0.01) lower n-6/n-3 PUFA ratio (3.73 vs. 11.40) as well as a lower thrombogenic index (0.92 vs. 1.08; *p* < 0.01). The other calculated indices did not differ significantly (*p* > 0.05) between the two groups. These indices did not vary between genders.

Table 5 displays the effects of dietary treatment and gender on the lipid oxidation of LTM, measured as the malondialdehyde (MDA) content.

Neither the diet nor gender affected (*p* > 0.05) the oxidative stability of fresh, refrigerated (8 days) or cooked LTM samples.

### 3.3. Fatty Acid Profile and Lipid Nutritional Indices of Subcutaneous Adipose Tissue and Perirenal Fat

Table 6 and Table 7 display the effects of dietary treatment and gender on fatty acid profile of the SF and PF, respectively.

The SFA content in SF did not vary (*p* > 0.05) with the treatments (Table 6), whereas it decreased (*p* < 0.05), mostly in stearic acid, in PF when the dietary n-6/n3 PUFA ratio (LPR group) is lowered (Table 7).

In terms of MUFA content, while observing the same trend in both tissues, lowering the dietary ratio of n-6/n-3 PUFA did not significantly affect (*p* > 0.05) the single components and the total MUFA contents in PF (Table 7); however, in SF (Table 6), this dietary modification resulted in a significant reduction (*p* < 0.01) in the total MUFA content, specifically affecting heptadecenoic, oleic (*p* < 0.01) and vaccenic (*p* < 0.05) fatty acids.

The total content of PUFA, as well as most of the individual PUFA in both Ats, were higher (*p* < 0.01) in the LPR group. In detail, the linoleic acid content increased in both ATs but was significantly higher only in PF (*p* < 0.01), while the n-3 PUFA, such as ALA, eicosatrienoic, EPA, DPA, the essential fatty acids (EFA) and the n-3 LCPUFA, were higher (*p* < 0.01) in both ATs of the LPR group. Furthermore, in this group, DHA and DFA were significantly higher (*p* < 0.01 and *p* < 0.05, respectively) only in PF (Table 7). The LPR group showed higher UFA content in SF (*p* < 0.05) and PF (*p* < 0.01). The low dietary n-6/n-3 PUFA ratio led to an increase in the IV in both ATs, but it was statistically significant (*p* < 0.01) only in PF.

Overall, gender did not affect the FA composition of both adipose tissues. However, barrows exhibited higher (*p* < 0.05) contents of both UFA and DFA in SF (Table 6).

Table 8 and Table 9 display the effects of dietary treatment and gender on LNI in SF and PF, respectively.

The data show that the LNI were significantly affected by dietary treatment in both ATs. In SF, the LPR group had higher UFA/SFA (*p* < 0.05), PUFA/SFA (*p* < 0.01) and H/H ratios, (*p* < 0.01), while the n-6/n-3 PUFA ratio was lower (*p* < 0.01) (Table 8). Additionally, HPI was significantly higher (*p* < 0.05) in the LPR group. This group showed lower values of TI (*p* < 0.01) and AI (*p* < 0.05). However, significantly higher (*p* < 0.01) values of UI and PI were observed in the LPR group. As already seen in LTM, gender did not influence these indices in subcutaneous adipose tissue (*p* > 0.05), except for UI, which was higher (*p* < 0.05) in barrows (Table 8). Regarding the lipid nutritional indices of PF (Table 9), the trend is similar to that observed for SF. In fact, the UFA/SFA, PUFA/SFA and H/H ratios were enhanced (*p* < 0.01) in the LPR group, while the n-6/n-3 PUFA ratio, AI and TI were lower (*p* < 0.01). Moreover, in this group, both UI and PI increased significantly (*p* < 0.01).

In PF, there was no difference (*p* > 0.05) between barrows and gilts in UFA/SFA, PUFA/SFA and n-6/n-3 ratios (Table 9). Barrows had higher H/H ratio (*p* < 0.05) and HPI (*p* < 0.01) compared to gilts, while the latter showed higher (*p* < 0.05) values of AI and TI.

**Figure 1:**
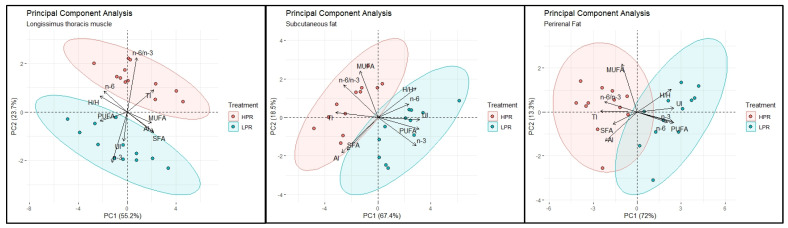
Principal Component Analysis. Biplot of the results of longissimus thoracis muscle (LTM), subcutaneous (SF) and perirenal (PF) adipose tissues (ATs). According to diet treatment, each plot discriminates the variables SFA: saturated fatty acids; MUFA: monounsaturated fatty acids; PUFA: polyunsaturated fatty acids; n-3 PUFA, n-6 PUFA, n-6/n-3 PUFA ratio, H/H: hypocholesterolemic/hypercholesterolemic ratio; AI: atherogenic index; TI: thrombogenic index; UI: unsaturation index.

In Figure 1, through Principal Component Analysis, a clear separation between the two dietary groups is graphically visualized for all the tissues examined.

The biplot graphs simultaneously show the distribution of samples in the plane described by the principal components and the influence of the studied variables on this same distribution. The farther the arrow of a variable moved away from the center, the more significant the impact of that variable has been. For example, the variable n6/n3 PUFA ratio influenced the description of the distribution plane of the HPR samples with higher values. Similarly, we can observe that the LPR samples were influenced by the high values of the total PUFA, n-3 and n-6 PUFA in the case of both adipose tissues. It can be noted that, as anticipated, the increase in PUFA in the LPR samples led to an increase in UI, which, as seen, influenced the distribution of these same samples in the plane of the principal components. Similarly, in the two adipose tissues, it is visible how the clustering of HPR samples was influenced by high values of MUFA, SFA, AI, and TI.

These distinctive patterns observed in the PCA plots suggest that the alterations in fatty acid composition and nutritional indices are not random occurrences. Rather, they are indicative of discernible trends closely linked to varying ratios of dietary n-6/n-3 polyunsaturated fatty acids (PUFAs). These trends underscore the significant impact of the experimental interventions on the biochemical composition of the investigated tissues. This observation reinforces the idea that the specific dietary manipulations exerted a notable influence on the metabolic profiles of the tissues, further emphasizing the importance of considering the role of dietary factors in shaping the biochemical landscape.

## 4. Discussion

### 4.1. Carcass Characteristics

The pigs’ carcass traits were not affected by different dietary n-6/n-3 PUFA ratios. This agrees with the findings of De Tonnac and Mourot who could not find any effect of varying the n-6/n-3 PUFA ratios on carcass characteristics, except for an increase in the weight of the liver [48]. Liu and Kim found that the different dietary n-6/n-3 PUFA ratios did not influence backfat thickness and lean meat percentage [49]. Feeding linseed or fish oil to raise the n-3 PUFA intake of growing–finishing pigs did not influence carcass parameters [50]. Furthermore, similar findings were reported in Heigai pigs fed diets supplemented with different n-6/n-3 PUFA ratios, although it was observed that the reduction from eight to five of the dietary n-6/n-3 PUFA ratio yielded lighter carcasses [51]. Such discrepancy may be accounted for by differences in the duration of the dietary treatment, the genetic type involved in the study, and the slaughter weight. In the present study, the gender did not influence the carcass traits. Our findings also aligned with those of other researchers [52,53] who have observed gender-related impacts neither on carcass weight nor intramuscular lipid content. However, it is worth noting that some studies have reported that barrows tend to yield heavier carcasses compared to gilts [54].

### 4.2. Lipid Characteristics

Many studies have highlighted that the characteristics of dietary fats influence the composition of lipids in swine tissues [55,56]. Our results demonstrate that lowering the dietary n-6/n-3 PUFA ratio from 9.7:1 to 1.4:1 alters the FA profile in LTM, SF and PF of medium–heavy pigs, thus confirming previous reports [51,57].

In agreement with a previous study [49], the different n-6/n-3 PUFA dietary ratios did not change the total contents of SFA, MUFA and PUFA in LTM. However, in adipose tissues, the low dietary n-6/n-3 PUFA ratio significantly increased PUFA contents. This is in accordance with previous studies [57,58], highlighting how the intramuscular fat of the LTM is comparatively less sensitive than adipose tissues to PUFA dietary incorporation. Moreover, it has been emphasized [59,60] that, in pigs, n-3 long-chain PUFA are synthesized from dietary ALA. The long-term dietary administration of ALA can significantly increase the accumulation of this same fatty acid in body phospholipids and can improve the efficiency of its conversion to longer chain n3-PUFA, namely, EPA and DPA [61]. In the tissues we studied, the low dietary n-6/n-3 PUFA ratio, containing higher percentages of ALA, enhanced the content of ALA and total n-3 PUFA, except DHA, whose content did not vary. The lack of DHA production from linolenic acid has already been demonstrated by many authors [62,63,64,65].

Consistent with another previous study [33], our results also showed no effect of the dietary n-6/n-3 PUFA ratios on total n-6 PUFA in muscle, whereas the arachidonic and docosatetraenoic acids were reduced. The low dietary n-6/n-3 PUFA ratio enhanced the relative content of n-3 PUFA and n-3 LCPUFA in the intramuscular fat and adipose tissues at the expense of arachidonic and docosatetraenoic acids [57]. Additionally, the essential fatty acids (EFA) contents in muscle and adipose tissues were improved in pigs fed the low dietary n-6/n-3 PUFA ratio.

In the perirenal fat, characterized by high concentrations of SFA, the low dietary n-6/n-3 PUFA ratio led to a reduction in the total SFA and, notably, stearic acid content.

In this study, the dietary n-6/n-3 PUFA ratio affected the MUFA content, particularly the oleic acid, in SF but not in LTM and PF. Oleic acid, the most abundant fatty acid in swine tissues [66], is partly provided by diet and is also synthesized from C18:0 by ∆ 9-desaturase (stearoyl-CoA-desaturase); the activity of this enzyme is higher in the subcutaneous adipose tissue than in perirenal or intramuscular fats [67]. The activity of the enzyme is inhibited by high linolenic acid dietary levels [63]. Eventually, this could partly explain the differences observed among tissues. As regards the LNI, we found that the n-6/n-3 PUFA ratio in LTM and ATs was positively correlated with its dietary ratio. In fact, the low dietary n-6/n-3 PUFA ratio reduced the n-6/n-3 PUFA ratio, which ranged from values above 9 to 3.73 in LTM, and 2.24 and 2.01 in SF and PF, respectively. Similar findings were obtained in previous studies [60,68] that found that the increased n-3 PUFA content in the diet decreased the n-6/n-3 PUFA ratio in pork.

Consuming diets with n-6/n-3 PUFA ratio below 4.0 reduces the occurrence of cardiovascular diseases in humans [69]. Therefore, since the tissues examined from LPR diets showed values beneath the suggested maximum threshold, under this profile they could be regarded as beneficial for human food consumption. Meat and meat products with a more balanced n-6/n-3 PUFA ratio, as requested by the consumer, can be marketed as healthier foods, with premium prices in the market. Additionally, regulations and labeling standards that promote a balanced n-6/n-3 PUFA ratio could drive industry innovation and research to improve animal nutrition and rearing practices.

The UFA/SFA and PUFA/SFA ratios are used to value the healthiness of fats for human consumption. A balanced intake of dietary PUFA/SFA is thought to be important in regulating serum cholesterol [70]. A ratio of PUFA/SFA greater than 0.45 is recommended in human diets to prevent some chronic diseases [71]. In our study, the low dietary n-6/n-3 PUFA ratio brought about an increase of this parameter in all tissues examined, though only in LTM the minimum suggested value was exceeded.

The H/H index expresses the relationship between hypocholesterolemic fatty acids (C18:1n-9 and PUFA) and hypercholesterolemic fatty acids (C12:0, C14:0 and C16:0). This index can be used to evaluate the cholesterolemic effect of dietary lipids [72]. In this study, the different dietary n-6/n-3 PUFA ratios did not affect the H/H index of LTM, while the low dietary n-6/n-3 PUFA ratio improved this parameter in both the ATs, mainly cause of an increase in PUFA deposition. The values of H/H observed in our study fall within the range indicated for meat and meat products in previous studies [73].

The atherogenic index (AI) is represented by the ratio between the sum of the main SFA, thought as proatherogenic (favoring the adhesion of lipids to the cells of the immunological and circulatory system), and the sum of the UFA, deemed antiatherogenic (inhibiting the aggregation of plaque and diminishing the level of cholesterol) [71]. The thrombogenic index (TI) is defined as the ratio between the prothrombogenic FA (C14:0, C16:0 and C18:0) and anti-thrombogenic FA (MUFA, n-6 PUFA, and n-3 PUFA). At increasing TI values, the tendency to form clots in blood vessels increases. Both AI and TI can be related to platelet aggregation [74]. Low values for AI and TI represent a protective potential for coronary artery health. In the present study, the low dietary n-6/n-3 PUFA ratio significantly reduced the AI value in SF and PF, as well as the TI values in all the tissues examined. The health-promoting index (HPI) was the inverse of AI, therefore showing reciprocal values against the AI.

The unsaturation index (UI) and peroxidazibility index (PI) are potential tools to evaluate the susceptibility to oxidation of a tissue. These two indices are inversely related to the shelf-life of meat and meat products [75] and are positively related to the protective potential for coronary artery disease [71]. In the present study, the unsaturation index values were lower in the LTM than in ATs. Further, no changes in oxidative stability were observed in the muscle at varying diets neither on fresh nor cooked meat. In SF and PF, both UI and PI increased at lowering the dietary n-6/n-3 PUFA ratio. However, the calculated IV was below 70 for both diets, the maximum limit set by the regulations for Italian ham production [47].

Regarding gender, the intramuscular FA composition was very similar in barrows and gilts. These results conflict with experimental outcomes [59,76] where barrows yielded more saturated intramuscular fat than gilts. In our study, carcass fatness and intramuscular fat were the same in both genders. In fact, many authors [76,77,78] have indicated that the difference in FA composition of intramuscular fat between genders was accounted for by the higher degree of intramuscular fat in barrows, given that the proportion of SFA increased with the fat content of the carcass. The similar FA composition of LTM could explain the close values of nutritional indices between genders. In subcutaneous adipose tissue, the barrows showed a higher UFA content and consequently a higher value of unsaturation index. Overall, gender did not influence lipid nutritional indices, though barrows showed higher values of H/H and HPI and lower values of AI and TI in perirenal fat.

## 5. Conclusions

Based on the results of this study, we inferred that a low dietary n-6/n-3 polyunsaturated fatty acid ratio led to higher n-3 PUFAs content and better n-6/n-3 PUFA ratio in all the tissues examined, as well as to improved lipid nutritional indices, particularly for subcutaneous and perirenal adipose tissues. These changes have potential beneficial effects on human health while preserving the technological properties of the lipids, in particular without exerting negative effects on the oxidative stability. Conversely, gender seemed to play only a marginal role. Furthermore, the carcass characteristics were adversely affected neither by the low dietary n-6/n-3 polyunsaturated fatty acid ratio nor by gender.

This study confirms dietary intervention as a powerful tool for the quality control and improvement of meat and meat products. Given that the meat from medium–heavy pigs is intended also for use in non-PDO cured products, it is essential to conduct additional research to confirm the oxidative stability of cured products and assess the residual content of n-3 polyunsaturated fatty acids at the end of the curing period.

## Figures and Tables

**Table 1:** Ingredients (%), proximate composition (% on dry matter (DM) basis) and fatty acid composition (% of total fatty acids) of the diets.

		HPR		LPR	
Ingredients		Growing Period	Finishing Period	Growing Period	Finishing Period
Extruded linseed	%	0.00	0.00	5.00	5.00
Barley meal	%	85.50	91.00	80.50	86.60
Soybean meal	%	11.00	5.50	11.00	5.00
L-Lysine	%	0.31	0.29	0.30	0.29
DL-Methionine	%	0.06	0.04	0.06	0.03
L-Threonine	%	0.05	0.04	0.05	0.03
Calcium carbonate	%	1.18	1.13	1.19	1.15
Dicalcium phosphate	%	1.00	1.00	1.00	1.00
Salt (NaCl)	%	0.40	0.40	0.40	0.40
Vit/min pre-mix ^1^	%	0.50	0.50	0.50	0.50
Proximate composition					
Dry Matter (DM)	%	88.30	89.50	88.60	89.80
On DM basis					
Digestible energy	MJ/kg	13.35	13.26	13.63	13.54
Crude protein	%	16.87	12.55	17.89	13.20
Crude fat	%	2.00	1.73	4.30	3.86
Crude fibre	%	4.76	4.50	4.91	5.08
Ashes	%	5.87	4.50	5.86	5.89
Fatty acid composition	(% of total FAs)				
C14:0 (myristic)	%	0.47	0.39	0.25	0.21
C16:0 (palmitic)	%	29.01	24.25	18.13	15.20
C16:1 (palmitoleic)	%	0.49	0.34	0.17	0.15
C18:0 (stearic)	%	2.03	1.51	4.00	3.18
C18:1n-9 (oleic)	%	14.92	13.50	20.60	18.12
C18:2n-6 (linoleic)	%	47.55	53.67	33.50	34.69
C18:3n-3 (α-linolenic)	%	4.77	5.70	22.83	28.02
C20:1 (eicosenoic)	%	0.74	0.64	0.53	0.41
n-6/n-3 PUFA ratio		9.97	9.42	1.47	1.24

HPR: high dietary n-6/n-3 PUFA ratio (9.7:1); LPR: low dietary n-6/n-3 PUFA ratio (1.4:1). Growing period, from 80 to 113 kg of live weight (LW); finishing period, from 113 kg LW to slaughter (150 kg LW). ^1^ Vitamin/mineral pre-mix: providing the following nutrients (per kg diet as-fed): vitamin A 15,000 IU; vitamin D3 2000 IU; vitamin E (α-tocopheryl acetate) 50 mg; vitamin K 2.5 mg; vitamin B1 2 mg; vitamin B2 5 mg; vitamin B5 15 mg; vitamin B6 4 mg; vitamin B12 0.036 mg; niacin 25 mg; folic acid 1 mg; biotin 0.15 mg; choline 346 mg; Cu 15 mg; Fe 150 mg; Mn 25 mg; Co 0.4 mg; I 1.5 mg; Zn 100 mg; Se 0.1 mg.

**Table 2:** Effect of dietary treatment and gender on carcass traits (the hot carcass weight was the covariate for the slaughter live weight).

	Dietary Treatment			Gender			
	HPR (*N* = 12)	LPR (*N* = 12)	*p*-Value	Gilts (*N* = 12)	Barrows (*N* = 12)	*p*-Value	R-MSE
Slaughter live weight (kg)	146.90	152.90	0.185	150.50	149.20	0.760	10.599
Hot carcass weight (kg)	127.10	126.90	0.866	126.80	127.30	0.695	2.549
Backfat thickness (mm)	30.79	34.63	0.241	31.93	33.49	0.630	7.696
Lean meat content (%)	51.48	49.73	0.244	50.97	50.24	0.620	3.517
IMF LTM (%)	1.63	1.57	0.755	1.48	1.72	0.245	0.490

HPR: high dietary n-6/n-3 PUFA ratio (9.7:1); LPR: low dietary n-6/n-3 PUFA ratio (1.4:1); R-MSE: root means square error. IMF LTM: intramuscular fat of longissimus thoracis muscle.

**Table 3:** Fatty acid composition (g/100 g of total lipids) of intramuscular lipids of the longissimus thoracis muscle from pigs receiving a diet with different n-6/n-3 PUFA ratio: effect of dietary treatment and gender.

	Dietary Treatment			Gender			
Fatty Acids (FAs)	HPR (*N* = 12)	LPR (*N* = 12)	*p*-Value	Gilts (*N* = 12)	Barrows (*N* = 12)	*p*-Value	R-MSE
C10:0 (capric)	0.08	0.08	0.82	0.07	0.08	0.07	0.019
C12:0 (lauric)	0.05	0.05	0.45	**0.04**	**0.05**	0.03	0.001
C14:0 (myristic)	0.80	0.84	0.60	0.75	0.88	0.05	0.150
C16:0 (palmitic)	15.69	15.73	0.97	14.90	16.52	0.08	2.103
C17:0 (heptadecanoic)	0.16	0.14	0.09	0.14	0.15	0.25	0.019
C18:0 (stearic)	8.54	8.52	0.97	8.16	8.89	0.17	1.225
C20:0 (eicosanoic)	0.10	0.10	0.97	0.09	0.10	0.13	0.014
C16:1 (palmitoleic)	2.17	2.07	0.54	**1.90**	**2.34**	0.01	0.384
C17:1 (heptadecenoic)	**0.20**	**0.16**	<0.01	0.18	0.18	0.63	0.021
C18:1n-7 (vaccenic)	2.81	2.73	0.58	**2.58**	**2.96**	0.02	0.377
C18:1n-9 (oleic)	26.32	25.13	0.46	24.32	27.12	0.09	3.786
C20:1 (eicosenoic)	0.42	0.44	0.58	**0.39**	**0.47**	0.03	0.078
C18:2n-6 (linoleic)	6.30	6.45	0.65	6.57	6.18	0.24	0.772
C18:3n-3 (α-linolenic)	**0.32**	**1.24**	<0.01	0.77	0.79	0.64	0.093
C18:3n-6 (γ-linolenic)	0.15	0.12	0.06	0.13	0.14	0.89	0.027
C20:2n-6 (eicosadienoic)	0.15	0.16	0.68	0.16	0.15	0.70	0.020
C20:3n-3 (eicosatrienoic)	**0.05**	**0.15**	<0.01	0.10	0.10	0.89	0.012
C20:4n-6 (arachidonic)	**2.36**	**1.89**	0.03	2.30	1.96	0.10	0.471
C20:5n-3 (eicosapentaenoic)	**0.10**	**0.38**	<0.01	0.25	0.23	0.16	0.042
C22:4n-6 (docosatetraenoic)	**0.35**	**0.20**	<0.01	0.30	0.26	0.07	0.057
C22:5n-3 (docosapentaenoic)	**0.30**	**0.53**	<0.01	0.45	0.38	0.07	0.085
C22:6n-3 (docosahexaenoic)	0.06	0.06	0.43	**0.07**	**0.05**	<0.01	0.013
∑ SFA	25.41	25.45	0.98	24.16	26.70	0.09	3.430
∑ MUFA	31.92	30.52	0.47	29.37	33.07	0.06	4.537
∑ PUFA	10.14	11.19	0.09	11.10	10.23	0.16	1.444
∑ UFA	42.05	41.71	0.83	40.47	43.30	0.08	3.721
∑ n-6 PUFA	9.32	8.83	0.37	9.46	8.69	0.16	1.288
∑ n-3 PUFA	**0.82**	**2.36**	<0.01	1.64	1.54	0.23	0.191
∑ n-3 LCPUFA	**0.50**	**1.12**	<0.01	0.87	0.75	0.06	0.137
∑ DFA	50.59	50.24	0.85	48.64	52.19	0.08	4.643
∑ EFA	9.12	9.71	0.28	9.77	9.06	0.19	1.266

HPR: high dietary n-6/n-3 polyunsaturated (PUFA) ratio (9.7:1); LPR: low dietary n-6/n-3 PUFA ratio (1.4:1); R-MSE: root means square error. SFA: saturated FAs; MUFA: monounsaturated FAs; PUFA: polyunsaturated FAs; UFA: unsaturated FAs (MUFA + PUFA); n-3 LCPUFA: n-3 long-chain PUFA (eicosatrienoic + eicosapentaenoic + docosapentaenoic + docosahexaenoic acids); DFA: desirable FAs (MUFA + PUFA + stearic acid); EFA: essential FAs (linoleic + α-linolenic + γ-linolenic + arachidonic acids).

**Table 4:** Lipid nutritional indices (mean values and R-MSE) of intramuscular lipids of longissimus thoracis muscle from pigs receiving a diet with different n-6/n-3 PUFA ratio: effect of dietary treatment and gender.

	Dietary Treatment			Gender			
Item	HPR (*N* = 12)	LPR (*N* = 12)	*p*-Value	Gilts (*N* = 12)	Barrows (*N* = 12)	*p*-Value	R-MSE
UFA/SFA ratio	1.66	1.66	0.88	1.68	1.64	0.38	0.131
PUFA/SFA ratio	0.41	0.46	0.29	0.47	0.39	0.09	0.107
n-6/n-3 PUFA ratio	**11.40**	**3.73**	<0.01	7.64	7.49	0.42	0.459
H/H ratio	2.22	2.21	0.92	2.27	2.16	0.16	0.178
HPI	2.23	2.21	0.72	2.27	2.18	0.23	0.178
AI	0.45	0.46	0.66	0.44	0.46	0.19	0.037
TI	**1.08**	**0.92**	<0.01	0.98	1.03	0.24	0.098
UI	59.56	61.60	0.18	60.15	61.01	0.56	3.514
PI	21.98	24.76	0.07	24.63	22.11	0.10	3.547

HPR: high n-6/n-3 PUFA ratio (9.7:1); LPR: low n-6/n-3 PUFA ratio (1.4:1). SFA: saturated fatty acids; MUFA: monounsaturated fatty acids; PUFA: polyunsaturated fatty acids; UFA: unsaturated fatty acids. H/H: hypocholesterolemic/hypercholesterolemic ratio = (cis-C18:1 + ΣPUFA)/(C12:0 + C14:0 + C16:0) [42]. HPI: health-promoting index = ΣUFA/[C12:0 + (4 × C14:0) + C16:0] [43]. AI: atherogenic index = [C12:0 + (4 × C14:0) + C16:0]/ΣUFA [44]. TI: thrombogenic index = (C14:0 + C16:0 + C18:0)/[(0.5 × ΣMUFA) + (0.5 × Σn-6 PUFA) + (3 × Σn-3 PUFA) + (Σn-3 PUFA/Σn-6 PUFA)] [44]. UI: unsaturation index = (% monoenoics × 1) + (% dienoics × 2) + (% trienoics × 3) + (% tetraenoics × 4) + (% pentaenoics × 5) + (% hexaenoics × 6) [45]. PI: peroxidizability index = (% monoenoics × 0.025) + (% dienoics × 1) + (% trienoics × 2) + (% tetraenoics × 4) + (% pentaenoics × 6) + (% hexaenoics × 8) [46].

**Table 5:** Effect of dietary treatment and gender on MDA (malondialdehyde) content (mg/kg) of raw (at 24 h and 8 days) and cooked longissimus thoracis muscle.

	Dietary Treatment			Gender			
	HPR (*N* = 12)	LPR (*N* = 12)	*p*-Value	Gilts (*N* = 12)	Barrows (*N* = 12)	*p*-Value	R-MSE
MDA at 24 h post-mortem	0.105	0.113	0.715	0.097	0.122	0.263	0.053
MDA at 8 days of storage	0.144	0.198	0.112	0.157	0.185	0.404	0.078
MDA cooked at 24 h post-mortem	0.406	0.495	0.182	0.458	0.443	0.822	0.156

HPR: high dietary n-6/n-3 PUFA ratio (9.7:1); LPR: low dietary n-6/n-3 PUFA ratio (1.4:1); R-MSE: root means square error.

**Table 6:** Fatty acid composition (g/100 g of total lipids) of subcutaneous adipose tissue from pigs receiving a diet with different n-6/n-3 PUFA ratio: effect of dietary treatment and gender.

	Dietary Treatment			Gender			
Fatty Acids (FAs)	HPR (*N* = 12)	LPR (*N* = 12)	*p*-Value	Gilts (*N* = 12)	Barrows (*N* = 12)	*p*-Value	R-MSE
C10:0 (capric)	0.07	0.07	0.88	**0.07**	**0.08**	0.03	0.008
C12:0 (lauric)	0.01	0.01	0.18	0.01	0.01	0.61	0.001
C14:0 (myristic)	1.32	1.30	0.59	1.31	1.32	0.75	0.071
C16:0 (palmitic)	24.74	24.08	0.19	24.39	24.43	0.93	1.162
C17:0 (heptadecanoic)	**0.37**	**0.29**	<0.01	0.32	0.34	0.25	0.056
C18:0 (stearic)	15.68	14.70	0.09	15.35	15.03	0.57	1.352
C20:0 (eicosanoic)	0.20	0.19	0.25	0.20	0.20	0.96	0.023
C16:1 (palmitoleic)	1.80	1.73	0.36	1.72	1.80	0.36	0.190
C17:1 (heptadecenoic)	**0.34**	**0.28**	<0.01	0.30	0.32	0.20	0.045
C18:1n-7 (vaccenic)	**2.34**	**2.19**	0.02	2.20	2.33	0.06	0.156
C18:1n-9 (oleic)	**37.92**	**35.95**	<0.01	36.50	37.38	0.20	1.583
C20:1 (eicosenoic)	0.87	0.78	0.09	0.79	0.87	0.19	0.136
C18:2n-6 (linoleic)	8.67	9.52	0.14	8.77	9.40	0.28	1.358
C18:3n-3 (α-linolenic)	**0.74**	**3.80**	<0.01	2.14	2.40	0.14	0.417
C18:3n-6 (γ-linolenic)	**0.17**	**0.15**	0.02	0.15	0.16	0.19	0.021
C20:2n-6 (eicosadienoic)	0.42	0.44	0.38	0.40	0.45	0.07	0.057
C20:3n-3 (eicosatrienoic)	**0.13**	**0.55**	<0.01	0.32	0.36	0.30	0.047
C20:4n-6 (arachidonic)	**0.23**	**0.18**	0.01	0.20	0.21	0.57	0.037
C20:5n-3 (eicosapentaenoic)	**0.01**	**0.05**	<0.01	0.02	0.03	0.30	0.001
C22:4n-6 (docosatetraenoic)	**0.10**	**0.08**	<0.01	0.08	0.09	0.22	0.013
C22:5n-3 (docosapentaenoic)	**0.09**	**0.21**	<0.01	0.14	0.15	0.19	0.018
C22:6n-3 (docosahexaenoic)	0.02	0.03	0.10	0.03	0.02	0.39	0.005
∑ SFA	42.46	40.72	0.09	41.71	41.48	0.82	2.408
∑ MUFA	**43.28**	**40.93**	<0.01	41.51	42.69	0.13	1.811
∑ PUFA	**10.56**	**14.99**	<0.01	12.27	13.29	0.19	1.823
∑ UFA	**53.84**	**55.93**	0.03	**53.79**	**55.98**	0.02	2.153
∑ n-6 PUFA	9.58	10.37	0.20	9.62	10.32	0.25	1.448
∑ n-3 PUFA	**0.98**	**4.63**	<0.01	2.65	2.96	0.11	0.452
∑ n-3 LCPUFA	**0.24**	**0.82**	<0.01	0.51	0.56	0.06	0.065
∑ DFA	69.52	70.63	0.15	**69.14**	**71.01**	0.02	1.777
∑ EFA	**9.80**	**13.65**	<0.01	11.27	11.18	0.22	1.746
IV	59.88	62.08	0.07	60.57	61.09	0.48	2.804

HPR: high dietary n-6/n-3 PUFA ratio (9.7:1); LPR: low dietary n-6/n-3 PUFA ratio (1.4:1); R-MSE: root means square error. SFA: saturated FAs; MUFA: monounsaturated FAs; PUFA: polyunsaturated FAs; UFA: unsaturated FAs (MUFA + PUFA); n-3 LCPUFA: n-3 long-chain PUFA (eicosatrienoic + eicosapentaenoic + docosapentaenoic + docosahexaenoic acids); DFA: desirable FAs (MUFA + PUFA + stearic acid); EFA: essential FAs (linoleic + α-linolenic + γ-linolenic + arachidonic. IV: iodine value = (85.703 + [C14:0] × 2.740 − [C16:0] × 1.085 − [C18:0] × 0.710 + [C18:2n-6] × 0.986) [47].

**Table 7:** Fatty acid composition (g/100 g of total lipids) of perirenal fat from pigs receiving a diet with different n-6/n-3 PUFA ratio: effect of dietary treatment and gender.

	Dietary Treatment			Gender			
Fatty Acids (FAs)	HPR (*N* = 12)	LPR (*N* = 12)	*p*-Value	Gilts (*N* = 12)	Barrows (*N* = 12)	*p*-Value	R-MSE
C10:0 (capric)	0.09	0.09	0.62	0.09	0.9	0.72	0.013
C12:0 (lauric)	0.09	0.09	0.27	0.09	0.09	0.30	0.009
C14:0 (myristic)	1.42	1.46	0.17	1.47	1.42	0.05	0.068
C16:0 (palmitic)	28.02	27.49	0.22	27.99	27.52	0.28	1.019
C17:0 (heptadecanoic)	0.36	0.31	0.20	0.34	0.34	0.92	0.080
C18:0 (stearic)	**22.56**	**21.35**	0.03	22.21	21.67	0.33	1.333
C20:0 (eicosanoic)	0.21	0.20	0.41	0.21	0.20	0.39	0.024
C16:1 (palmitoleic)	1.35	1.26	0.23	1.31	1.31	0.98	0.175
C17:1 (heptadecenoic)	0.24	0.20	0.07	0.21	0.23	0.38	0.058
C18:1n-7 (vaccenic)	1.62	1.51	0.13	1.57	1.57	1.00	0.158
C18:1n-9 (oleic)	30.29	28.71	0.09	28.85	30.16	0.15	2.139
C20:1 (eicosenoic)	0.53	0.48	0.14	0.48	0.53	0.19	0.083
C18:2n-6 (linoleic)	**6.92**	**8.76**	<0.01	7.88	7.79	0.89	1.473
C18:3n-3 (α-linolenic)	**0.63**	**4.00**	<0.01	2.23	2.41	0.14	0.307
C18:3n-6 (γ-linolenic)	0.13	0.12	0.28	0.12	0.13	0.39	0.017
C20:2n-6 (eicosadienoic)	**0.24**	**0.29**	<0.01	0.26	0.27	0.54	0.038
C20:3n-3 (eicosatrienoic)	**0.07**	**0.40**	<0.01	0.22	0.25	0.14	0.036
C20:4n-6 (arachidonic)	0.20	0.17	0.06	0.19	0.18	0.40	0.040
C20:5n-3 (eicosapentaenoic)	**0.01**	**0.05**	<0.01	0.03	0.03	0.21	0.009
C22:4n-6 (docosatetraenoic)	**0.08**	**0.06**	<0.01	0.07	0.07	0.85	0.010
C22:5n-3 (docosapentaenoic)	**0.07**	**0.20**	<0.01	0.13	0.14	0.48	0.022
C22:6n-3 (docosahexaenoic)	**0.02**	**0.03**	0.02	0.03	0.02	0.13	0.005
∑ SFA	**52.76**	**50.97**	0.04	52.41	51.31	0.21	2.026
∑ MUFA	34.04	32.17	0.07	32.42	33.80	0.17	2.350
∑ PUFA	**8.37**	**14.08**	<0.01	11.16	11.30	0.86	1.834
∑ UFA	**42.42**	**46.25**	<0.01	43.57	45.09	0.13	2.307
∑ n-6 PUFA	**7.57**	**9.40**	<0.01	8.53	8.44	0.90	1.559
∑ n-3 PUFA	**0.81**	**4.68**	<0.01	2.63	2.85	0.14	0.358
∑ n-3 LCPUFA	**0.18**	**0.68**	<0.01	0.41	0.44	0.23	0.061
∑ DFA	**64.98**	**67.57**	<0.01	65.79	66.76	0.30	2.200
∑ EFA	**7.87**	**13.05**	<0.01	10.41	10.51	0.89	1.755
IV	**49.99**	**53.38**	<0.01	51.36	52.02	0.57	2.732

HPR: high dietary n-6/n-3 PUFA ratio (9.7:1); LPR: low dietary n-6/n-3 PUFA ratio (1.4:1); R-MSE: root means square error. SFA: saturated FAs; MUFA: monounsaturated FAs; PUFA: polyunsaturated FAs; UFA: unsaturated FAs (MUFA + PUFA); n-3 LCPUFA: long-chain PUFA (eicosatrienoic + eicosapentaenoic + docosapentaenoic + docosahexaenoic acids); DFA: desirable FAs (MUFA + PUFA + stearic acid); EFA: essential FAs (linoleic + α-linolenic + γ-linolenic + arachidonic acids). IV: iodine value = (85.703 + [C14:0] × 2.740 − [C16:0] × 1.085 − [C18:0] × 0.710 + [C18:2n-6] × 0.986) [47].

**Table 8:** Lipid nutritional indices (mean values and R-MSE) of subcutaneous adipose tissue from pigs receiving different n-6/n-3 PUFA ratio: effect of dietary treatment and gender.

	Dietary Treatment			Gender			
Item	HPR (*N* = 12)	LPR (*N* = 12)	*p*-Value	Gilts (*N* = 12)	Barrows (*N* = 12)	*p*-Value	R-MSE
UFA/SFA ratio	**1.27**	**1.38**	0.03	1.29	1.36	0.18	0.110
PUFA/SFA ratio	**0.25**	**0.37**	<0.01	0.30	0.32	0.23	0.054
n-6/n-3 PUFA ratio	**9.55**	**2.24**	<0.01	5.90	5.89	0.45	0.192
H/H ratio	**1.86**	**2.01**	0.01	1.90	1.97	0.21	0.136
HPI	**1.80**	**1.91**	0.03	1.82	1.89	0.18	0.126
AI	**0.56**	**0.53**	0.04	0.55	0.53	0.22	0.037
TI	**1.42**	**1.01**	<0.01	1.25	1.18	0.14	0.108
UI	**66.48**	**76.80**	<0.01	**69.84**	**73.44**	0.04	4.027
PI	**14.31**	**22.74**	<0.01	17.80	19.26	0.16	2.434

HPR: high n-6/n-3 PUFA ratio (9.7); LPR: low n-6/n-3 PUFA ratio (1.4). SFA: saturated fatty acids; MUFA: monounsaturated fatty acids; PUFA: polyunsaturated fatty acids; UFA: unsaturated fatty acids. H/H: hypocholesterolemic/hypercholesterolemic ratio = (cis-C18:1 + ΣPUFA)/(C12:0 + C14:0 + C16:0) [42]. HPI: Health-Promoting Index = ΣUFA/[C12:0 + (4 × C14:0) + C16:0] [43]. AI: Atherogenic Index = [C12:0 + (4 × C14:0) + C16:0]/ΣUFA [44]. TI: Thrombogenic Index = (C14:0 + C16:0 + C18:0)/[(0.5 × ΣMUFA) + (0.5 × Σn-6 PUFA) + (3 × Σn-3 PUFA) + (Σn-3 PUFA/Σn-6 PUFA)] [44]. UI: Unsaturation Index = (% monoenoics × 1) + (% dienoics × 2) + (% trienoics × 3) + (% tetraenoics × 4) + (% pentaenoics × 5) + (% hexaenoics × 6) [45]. PI: Peroxidizability Index = (% monoenoics × 0.025) + (% dienoics × 1) + (% trienoics × 2) + (% tetraenoics × 4) + (% pentaenoics × 6) + (% hexaenoics × 8) [46].

**Table 9:** Lipid nutritional indices (mean values and R-MSE) of perirenal fat from pigs receiving a diet with different n-6/n-3 PUFA ratio: effect of dietary treatment and gender.

	Dietary Treatment			Gender			
Item	HPR (*N* = 12)	LPR (*N* = 12)	*p*-Value	Gilts (*N* = 12)	Barrows (*N* = 12)	*p*-Value	R-MSE
UFA/SFA ratio	**0.80**	**0.91**	<0.01	0.83	0.88	0.07	0.059
PUFA/SFA ratio	**0.16**	**0.28**	<0.01	0.21	0.22	0.68	0.040
n-6/n-3 PUFA ratio	**9.14**	**2.01**	<0.01	5.64	5.52	0.27	0.256
H/H ratio	**1.31**	**1.47**	<0.01	**1.35**	**1.43**	0.03	0.076
HPI	**1.26**	**1.38**	<0.01	**1.28**	**1.36**	<0.01	0.071
AI	**0.80**	**0.73**	<0.01	**0.78**	**0.74**	0.02	0.040
TI	**2.23**	**1.43**	<0.01	**1.88**	**1.77**	0.04	0.123
UI	**52.52**	**66.18**	<0.01	58.41	60.29	0.25	3.807
PI	**11.47**	**21.54**	<0.01	16.31	16.70	0.70	2.364

HPR: high n-6/n-3 PUFA ratio (9.7:1); LPR: low n-6/n-3 PUFA ratio (1.4:1). SFA: saturated fatty acids; MUFA: monounsaturated fatty acids; PUFA: polyunsaturated fatty acids; UFA: unsaturated fatty acids. H/H: hypocholesterolemic/hypercholesterolemic ratio = (cis-C18:1 + ΣPUFA)/(C12:0 + C14:0 + C16:0) [42]. HPI: health-promoting index = ΣUFA/[C12:0 + (4 × C14:0) + C16:0] [43]. AI: atherogenic index = [C12:0 + (4 × C14:0) + C16:0]/ΣUFA [44]. TI: thrombogenic index = (C14:0 + C16:0 + C18:0)/[(0.5 × ΣMUFA) + (0.5 × Σn-6 PUFA) + (3 × Σn-3 PUFA) + (Σn-3 PUFA/Σn-6 PUFA)] [44]. UI: unsaturation index = (% monoenoics × 1) + (% dienoics × 2) + (% trienoics × 3) + (% tetraenoics × 4) + (% pentaenoics × 5) + (% hexaenoics × 6) [45]. PI: peroxidizability index = (% monoenoics × 0.025) + (% dienoics × 1) + (% trienoics × 2) + (% tetraenoics × 4) + (% pentaenoics × 6) + (% hexaenoics × 8) [46].

## Data Availability

The data used to support the findings of this study can be made available by the corresponding author upon request.

## Abbreviations

AI	Atherogenic index
ALA	Alpha-linolenic acid
ATs	Adipose tissues
CVD	Cardiovascular diseases
DFA	Desirable fatty acids
DHA	Docosahexaenoic acid
DM	Dry matter
DPA	Docosapentanoic acid
EFA	Essential fatty acids
EPA	Eicosapentaenoic acid
EVOH	Ethylene vinyl alcohol
FA	Fatty acid
FAMEs	Fatty acids methyl esters
GC	Gas chromatography
GLA	γ-linolenic acid
GLM	General linear model
H/H	Hypocholesterolemic/hypercholesterolemic ratio
HPI	Health-Promoting Index
HPR	High dietary n-6/n-3 polyunsaturated fatty acid ratio
IMF	Intramuscular fat
IV	Iodine value
LA	Linoleic acid
LCPUFA	Long-chain polyunsaturated fatty acids
LDL	Low density lipoproteins
LNI	Lipid nutritional indices
LPR	Low dietary n-6/n-3 polyunsaturated fatty acid ratio
LTM	Longissimus thoracis muscle
LW	Live weight
MAP	Modified atmosphere packaging
MDA	Malondialdehyde
MUFA	Monounsaturated fatty acids
OTR	Oxygen transmission rate
PCA	Principal component analysis
PDO	Protected denomination of origin
PE	Polyethylene
PET	Polyethylene terephthalate
PF	Perirenal fat
PI	Peroxidizability index
PR	n-6/n-3 polyunsaturated fatty acids ratio
PUFA	Polyunsaturated fatty acids
R-MSE	Root means square error
SF	Subcutaneous fat
SFA	Saturated fatty acids
TBARS	2-Thiobarbituric acid reactive substances
TEP	Tetraethoxypropane
TI	Thrombogenic index
UFA	Unsaturated fatty acids
UI	Unsaturation index
